# Ophthalmic complications in retinopathy of prematurity in the first decade of life in Korea using the national health insurance database

**DOI:** 10.1038/s41598-021-04616-7

**Published:** 2022-01-18

**Authors:** Eun Hee Hong, Yong Un Shin, Gi Hwan Bae, Young Jin Choi, Seong Joon Ahn, Inah Kim, Heeyoon Cho

**Affiliations:** 1 Department of Ophthalmology, Hanyang University College of Medicine, Seoul, Korea; 2 Department of Occupational and Environment Medicine, Hanyang University College of Medicine, Seoul, Korea; 3 Department of Pediatrics, Hanyang University College of Medicine, Seoul, Korea

**Keywords:** Retinal diseases, Vision disorders, Risk factors, Epidemiology, Preterm birth, Retinopathy of prematurity

## Abstract

The aim of this study is to investigate the epidemiology of ophthalmic complications of retinopathy of prematurity (ROP) after preterm birth using population-based database in South Korea. Using the National Health Insurance database, ophthalmic complications among premature infants born in 2007–2008 during their 10-year follow-up period were identified. Annual cumulative incidence rate and period prevalence of complications at each age were analyzed among those with ROP and those who underwent treatment for ROP (tROP). The hazard ratios (HRs) according to the presence of ROP and treatment for ROP were also analyzed. We identified 18,256 premature infants, 6995 of whom had ROP. The prevalence at 10th year for overall ophthalmic complications was 11.1% and 35.9% among ROP and tROP, respectively. Strabismus, amblyopia, and glaucoma were the three most common complications. The presence of ROP was associated with higher risk of complications (HR 1.53, 95%CI 1.44–1.61) among premature infants, and the presence of treatment for ROP was associated with higher risk of complications (HR 4.31, 95%CI 3.74–4.98) among ROP cases. This study reports the nationwide epidemiologic data on ophthalmic complications of ROP during the first decade of life, which will help advance our understandings and establish national strategies in managing ROP.

## Introduction

Retinopathy of prematurity (ROP) is characterized by abnormal growth of retinal blood vessels in the eyes of premature infants due to an arrest of normal retinal neuronal and vascular development^1,2^. It can have varying degrees of impact on long-term visual function, with blindness in the most severe cases^3,4^. With the improvement of care for premature infants, the survival rate of premature infants has increased. Accordingly, there has been a growing interest in complications after preterm birth^5^. ROP is one of the most important complications of preterm birth. Therefore, complications that may appear after ROP are also of great concern in children born prematurely^5^. Reported ophthalmic complications after ROP include early or late retinal detachment (RD), cataract, glaucoma, strabismus, refractive problems, amblyopia, and nystagmus^6–8^.

The development and impact of these ophthalmic complications according to the presence or severity of ROP were reported in previous studies^9–13^. However, the epidemiologic features of these complications in ROP in the nationwide population or during long-term follow-up have rarely been reported. We used the National Health Insurance (NHI) database in South Korea to investigate the 10-year outcomes of these ocular complications in ROP for the first time. The NHI database includes information on the total population, such as information on disease diagnosis and treatment in the entire population, to allow researchers to obtain population-based epidemiological data. The current study aimed to (1) investigate the nationwide prevalence and incidence of ophthalmic complications in ROP throughout the first decade of life, and (2) analyze the risk of developing ophthalmic complications according to the presence of ROP or treatment for ROP in South Korea.

## Methods

The Institutional Review Board (IRB) of Hanyang University Guri Hospital (Gyunggi-do, South Korea) approved this nationwide population-based retrospective cohort study (IRB File no. 2018-04-001) and waived the requirement for written informed consent because of the retrospective design. The study was conducted in accordance with the tenets of the Declaration of Helsinki.

### Database

Health claims data recorded from 2007 to 2018 in the Health Insurance Review and Assessment (HIRA) service of South Korea was used. The Korean National Health Insurance Service (KNHIS), the single national insurance provider, is compulsory health insurance covering > 97% of the population in South Korea. The remaining Korean population not insured by the KNHIS is covered by the Medical Care for Patriots and Veterans Affairs Scheme or the Medical Assistance Program^14^. All these claims and medical expenses are reviewed by HIRA, and the HIRA database contains data on demographic information, direct medical costs, diagnoses, procedures, and prescription records. In South Korea, there is a unique identification number system in which all Korean residents are assigned a unique number (Korean Resident Registration Number) at the time of birth, so patients in the HIRA database are identified by this identification number. Taken together, all medical claims made in South Korea are included in the HIRA database and can be used without duplication or omission.

### Cohort and case definition

In this study, the Korean Classification of Disease, sixth edition, which was adapted for the Korean healthcare system based on the International Classification of Diseases, 10th edition (ICD-10), was used to identify cases from the HIRA database. The diagnostic codes of “Extreme immaturity (P07.2, gestational age (GA) < 28 weeks)” and “Other preterm infants (P07.3, 28 weeks ≤ GA < 37 weeks)” were used to identify preterm infants with a GA of < 37 weeks who were born between 2007 and 2008. Cases with congenital anomalies and perinatal injuries that might affect normal development were excluded using diagnostic codes as described in Supplementary Table S1.

The diagnosis of ROP was based on the diagnostic code of ROP (H351) within 180 days of the diagnosis of preterm infants (P07.2-3). Among those with ROP, patients who underwent treatment for ROP (tROP) were identified using the procedure codes of pars plana vitrectomy (S5121-2), RD surgery (S5130), retinal photocoagulation (S5160), or cryopexy (S5140), within 1 year after the diagnosis of ROP.

### Epidemiology of ophthalmic complications in patients with ROP

Ophthalmic complications were identified using diagnostic codes of amblyopia, cataract, glaucoma, nystagmus, strabismus, and RD as described in Supplementary Table S2. Patients with ROP who had at least one diagnosis of these complications were included in the total ophthalmic complication group. We further identified patients who underwent cataract surgery in the total ophthalmic complication group using procedure codes as described in Supplementary Table S2. We also investigated cases with “refractive abnormalities,” using the diagnostic codes as described in Supplementary Table S2, which were not included in the total ophthalmic complications.

The annual period prevalence and annual cumulative incidence of each complication at each age after birth were calculated for patients with ROP and tROP. Hazard ratios (HRs) of total and each ophthalmic complication were analyzed according to (1) the presence of ROP among preterm infants (premature infants with ROP vs. premature infants without ROP) and (2) the performance of ROP treatment (ROP infants who underwent treatment for ROP vs. ROP infants who did not undergo treatment for ROP).

### Statistical analysis

The annual period prevalence and annual cumulative incidence rate at each age were calculated by dividing the number of prevalent and incident cases by the population. The Cox proportional hazards regression model was used to calculate HRs and 95% confidence intervals (CIs). The model included sex (male vs. female), income level of the patient's household (insurance payment classes: low vs. middle, high), area of residence (metropolitan cities vs. others), and year of prematurity diagnosis (2007 vs. 2008). Statistical significance was set at *p* < 0.05. All analyses were conducted using SAS version 9.4 (SAS Inc., Cary, NC).

## Results

### Patient demographics

**Figure 1:**
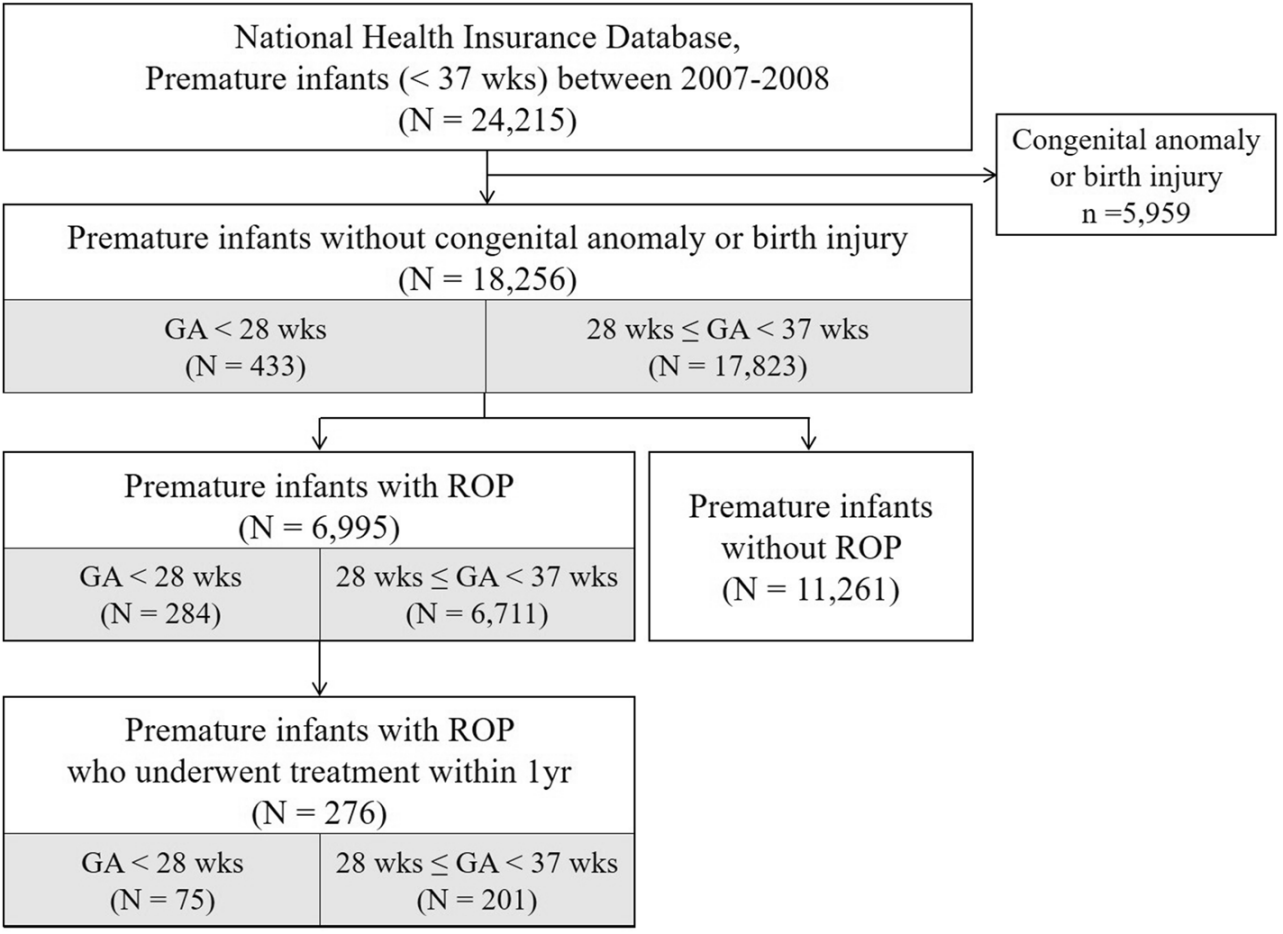
The enrollment of study patients. Premature infants with a gestational age (GA) < 37 weeks who were born between 2007 and 2008 were enrolled.

In total, 24,215 premature infants with a GA < 37 weeks were identified in South Korea between January 1, 2007 and December 31, 2008. Of them, 5959 patients with congenital anomalies or prenatal injuries were excluded. The remaining 18,256 premature infants were followed-up for 10 years. Figure 1 shows the enrollment of the study participants. In the study population, 6995 premature infants were diagnosed with ROP, and 276 of them underwent treatment for ROP. The number of premature infants without ROP was 11,261.

### Annual prevalence and incidence of ophthalmic complications in ROP

**Figure 2:**
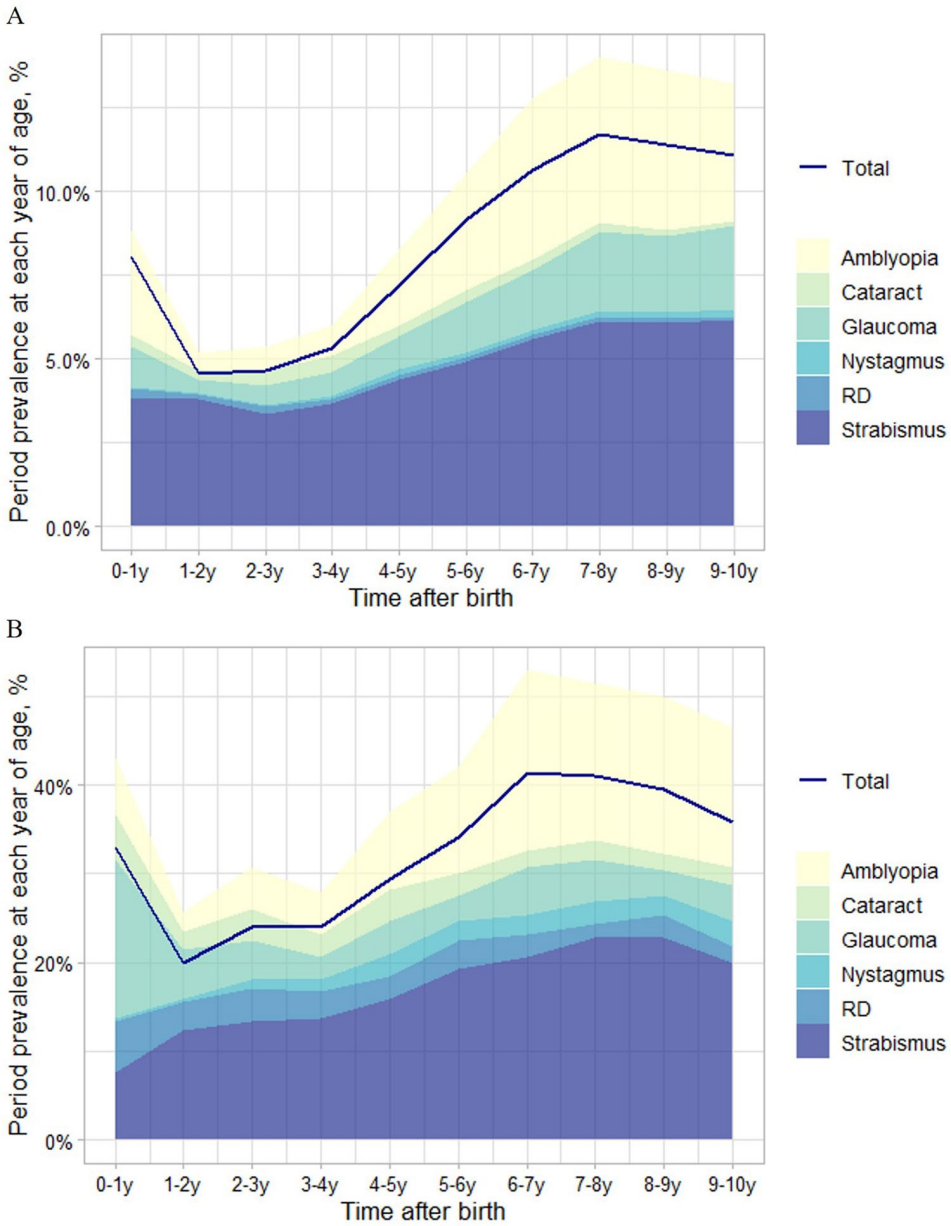
The period prevalence of ophthalmic complications at each year of life among patients with ROP (**A**) and those treated for ROP (**B**). “Total” represents the cases with at least one of the complications (amblyopia, cataract, glaucoma, nystagmus, retinal detachment (RD), and strabismus). ROP, retinopathy of prematurity.

At the 10th year of follow-up, ophthalmic complications were found in 11.1% of the ROP patients who were born at a GA < 37 weeks (19.0% in GA < 28 weeks; 10.7% in 28 weeks ≤ GA < 37 weeks) and 35.9% of the tROP patients who were born at a GA < 37 weeks (42.7% in GA < 28 weeks; 33.3% in 28 weeks ≤ GA < 37 weeks, Table 1). Table 2 and Fig. 2 show the annual period prevalence of total and each ophthalmic complication at each year of life among the infants with ROP and tROP. In the 10th year of follow-up, 4.09%, 0.13%, 2.50%, 0.23%, 6.15%, and 0.10% of the infants with ROP were diagnosed with amblyopia, cataract, glaucoma, nystagmus, strabismus, and RD, respectively. Among patients with tROP, the prevalence of each complication at the 10th year was 15.6%, 2.17%, 3.99%, 2.90%, 19.9%, and 1.81%, respectively.

**Table 1:** The number of prevalent cases and period prevalence of ophthalmic complications in ROP and those treated for ROP (tROP) at each year of life according to the GA.

	Overall (GA < 37wks)				GA < 28wks				28wks ≤ GA < 37wks			
	ROP		tROP		ROP		tROP		ROP		tROP	
Total, N	6995		276		284		75		6711		201	

	Prevalent cases, N	Period prevalence, %	Prevalent cases, N	Period prevalence, %	Prevalent cases, N	Period prevalence, %	Prevalent cases, N	Period prevalence, %	Prevalent cases, N	Period prevalence, %	Prevalent cases, N	Period prevalence, %
Age												
~ 1 years	561	8.02%	91	32.97%	34	11.97%	13	17.33%	527	7.85%	78	38.81%
1–2 years	320	4.57%	55	19.93%	21	7.39%	10	13.33%	299	4.46%	45	22.39%
2–3 years	324	4.63%	66	23.91%	28	9.86%	18	24.00%	296	4.41%	48	23.88%
3–4 years	371	5.30%	66	23.91%	36	12.68%	17	22.67%	335	4.99%	49	24.38%
4–5 years	503	7.19%	81	29.35%	39	13.73%	22	29.33%	464	6.91%	59	29.35%
5–6 years	639	9.14%	94	34.06%	43	15.14%	28	37.33%	596	8.88%	66	32.84%
6–7 years	743	10.62%	114	41.30%	53	18.66%	30	40.00%	690	10.28%	84	41.79%
7–8 years	817	11.68%	113	40.94%	50	17.61%	30	40.00%	767	11.43%	83	41.29%
8–9 years	796	11.38%	109	39.49%	54	19.01%	30	40.00%	742	11.06%	79	39.30%
9–10 years	774	11.07%	99	35.87%	54	19.01%	32	42.67%	720	10.73%	67	33.33%

*GA*, Gestational age; *ROP*, retinopathy of prematurity.

**Table 2:** The number of prevalent cases and period prevalence of ophthalmic complications in patients with ROP and those treated for ROP (tROP) at each year of life.

	Amblyopia				Cataract				Glaucoma				Nystagmus			
	ROP		tROP		ROP		tROP		ROP		tROP		ROP		tROP	
Total, N	6995		276		6995		276		6995		276		6995		276	

Age	N*	%^#^	N*	%^#^	N*	%^#^	N*	%^#^	N*	%^#^	N*	%^#^	N*	%^#^	N*	%^#^
~ 1 years	219	3.13%	18	6.52%	27	0.39%	14	5.07%	83	1.19%	49	17.75%	3	0.04%	1	0.36%
1–2 years	38	0.54%	6	2.17%	17	0.24%	6	2.17%	28	0.40%	15	5.43%	4	0.06%	1	0.36%
2–3 years	51	0.73%	13	4.71%	31	0.44%	10	3.62%	39	0.56%	12	4.35%	6	0.09%	3	1.09%
3–4 years	63	0.90%	13	4.71%	35	0.50%	7	2.54%	48	0.69%	7	2.54%	7	0.10%	4	1.45%
4–5 years	159	2.27%	24	8.70%	21	0.30%	10	3.62%	71	1.02%	10	3.62%	11	0.16%	7	2.54%
5–6 years	242	3.46%	33	11.96%	27	0.39%	7	2.54%	104	1.49%	8	2.90%	10	0.14%	6	2.17%
6–7 years	338	4.83%	56	20.29%	21	0.30%	5	1.81%	124	1.77%	15	5.43%	11	0.16%	6	2.17%
7–8 years	345	4.93%	49	17.75%	17	0.24%	6	2.17%	166	2.37%	13	4.71%	14	0.20%	7	2.54%
8–9 years	332	4.75%	49	17.75%	14	0.20%	5	1.81%	159	2.27%	8	2.90%	12	0.17%	6	2.17%
9–10 years	286	4.09%	43	15.58%	9	0.13%	6	2.17%	175	2.50%	11	3.99%	16	0.23%	8	2.90%

	Strabismus				Retinal detachment				Refractive abnormalities							
	ROP		tROP		ROP		tROP		ROP		tROP					
Total, N	6995		276		6995		276		6995		276					

Age	N*	%^#^	N*	%^#^	N*	%^#^	N*	%^#^	N*	%^#^	N*	%^#^				
~ 1 years	268	3.83%	21	7.61%	20	0.29%	16	5.80%	78	1.12%	11	3.99%				
1–2 years	265	3.79%	34	12.32%	11	0.16%	9	3.26%	134	1.92%	21	7.61%				
2–3 years	236	3.37%	37	13.41%	13	0.19%	10	3.62%	217	3.10%	34	12.32%				
3–4 years	256	3.66%	38	13.77%	10	0.14%	8	2.90%	484	6.92%	55	19.93%				
4–5 years	307	4.39%	44	15.94%	9	0.13%	7	2.54%	745	10.65%	66	23.91%				
5–6 years	343	4.90%	53	19.20%	11	0.16%	9	3.26%	972	13.90%	73	26.45%				
6–7 years	390	5.58%	57	20.65%	9	0.13%	7	2.54%	1212	17.33%	68	24.64%				
7–8 years	429	6.13%	63	22.83%	8	0.11%	4	1.45%	1550	22.16%	80	28.99%				
8–9 years	426	6.09%	63	22.83%	9	0.13%	7	2.54%	1732	24.76%	81	29.35%				
9–10 years	430	6.15%	55	19.93%	7	0.10%	5	1.81%	1726	24.67%	94	34.06%				

*ROP*, retinopathy of prematurity.

*N, Prevalent cases; ^#^%, Period prevalence.

The annual incident cases and annual incidence rates of total and each ophthalmic complication at each year of life among patients with ROP and tROP are shown in Supplementary Tables S3-S4 and Supplementary Figure S1. Note that this incidence reflects the time of registration of diagnostic codes. Thus, it may not directly reflect the actual incidence of complications as the time of registration may differ from the time of the actual disease onset.

Among patients with ROP or tROP who developed any of the complications, the total number of patients who underwent cataract surgery was 14 (13 cases in the first year, one case at the age of 6 years; Supplementary Table S4), indicating that 5.07% (14/276) of the patients with tROP underwent cataract surgery during the 10-year follow-up.

### HRs of ophthalmic complications

**Figure 3:**
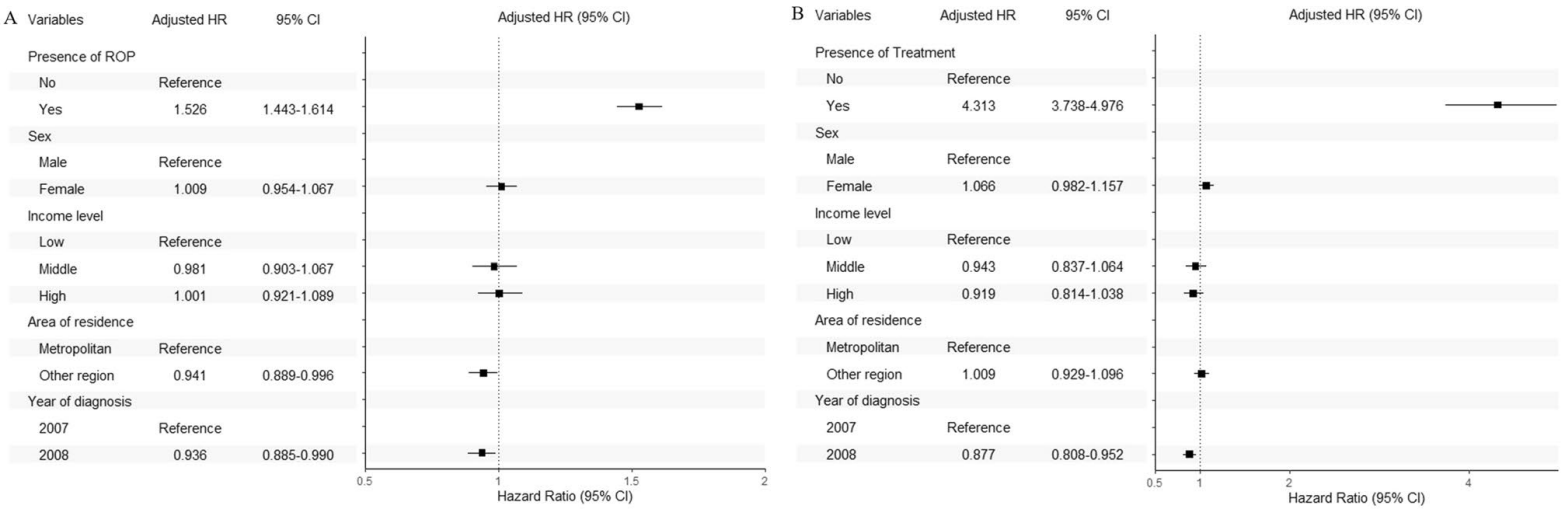
The hazard ratio plots of ophthalmic complications according to the presence of ROP among premature infants (**A**) and treatment for ROP among ROP infants (**B**). ROP, retinopathy of prematurity.

Among premature infants with GA < 37 weeks, those with ROP had a 1.526 times higher risk of ophthalmic complications (amblyopia, HR 1.607; cataract, HR 1.333; glaucoma, HR 1.363; nystagmus, HR 2.852; strabismus, HR 1.607; RD, HR 12.633; Fig. 3 and Supplementary Figure S2) after adjusting for sex, income level, area of residence, and year of diagnosis. Among premature infants with GA < 37 weeks and ROP, those who underwent treatment had a 4.313 times higher risk of ophthalmic complications (amblyopia, HR 3.640; cataract, HR 9.288; glaucoma, HR 4.609; nystagmus, HR 20.155; strabismus, HR 3.376; RD, HR 63.049; Fig. 3 and Supplementary Figure S3).

### Refractive abnormalities in ROP

The annual period prevalence of refractive abnormalities (myopia or hyperopia) at each year of life among patients with ROP and tROP are presented in Supplementary Figure S4. At the 10th year of follow-up, 24.7% of the patients with ROP and 34.1% of those with tROP had diagnostic codes of myopia or hyperopia (Table 2). Among premature infants with GA < 37 weeks, those with ROP had a 1.109 times higher risk of myopia or hyperopia. Among patients with ROP, those who underwent treatment had a 1.740 times higher risk of myopia or hyperopia.

## Discussion

The present study is the first nationwide epidemiological study of ophthalmic complications in patients with ROP. Our results provide informative data regarding the prevalence of ophthalmic complications during the first decade of life in ROP infants, as well as a comparison according to the presence of ROP and treatment for ROP. The three most common ophthalmic complications during the first 10 years were strabismus, amblyopia, and glaucoma. At the 10th year after birth, 11.1% of the cases with ROP and 35.9% of the cases with tROP showed ophthalmic complications. Among premature infants with a GA < 37 weeks, those with ROP had a 1.53 times higher risk of ophthalmic complications, among which those who underwent treatment had a 4.31 times higher risk of ophthalmic complications.

ROP is still one of the most important complications of preterm birth, even though it can be prevented by controlling the oxygen saturation level^15^. Although the prevalence of ROP is decreasing in some parts of the world with advances in controlling the environmental conditions of premature infants, ROP is reported to have an increasing incidence in some parts of the world and is considered a significant health problem^3,4,16^. Therefore, it is also important to understand the epidemiology of complications following ROP. Studies have reported the prevalence of various ophthalmic complications in ROP in Western countries. Thus far, the largest study of its kind was the Early Treatment of Retinopathy of Prematurity (ETROP) study^7,17–20^. The study included ROP infants with birth weight (BW) ≤ 1251 g born between October 1, 2000 and September 30, 2002 in multi-centers in the United States. The study reported that the prevalence of cataract at 6 months of age was 1.9%^20^, and that of glaucoma, RD, and strabismus at 6 years of age was 1.67%^19^, 16.2%^18^, and 42.2%^17^, respectively. The prevalence of nystagmus among bilateral high-risk prethreshold ROP was 22%^7^. In the present study, the prevalence of cataracts in the 1st year was 0.39%, and that of glaucoma, RD, and strabismus in the 6th year was 1.49%, 0.16%, and 4.90%, respectively among the ROP cases. All the prevalence rates herein were lower than those reported previously. This may be partially attributed to the study population. We could not use the diagnostic codes for BW in identifying premature infants. As the definition of “BW of less than 2500 g” is usually used for the diagnosis of premature infants, the inclusion of infants with BW ≤ 1251 g in the ETROP study should have led to the enrollment of more severe cases compared to that in the present study. Additionally, in the present study, the complications tended to show the highest prevalence at approximately 6 to 7 years of age. In South Korea, children enter elementary school at the age of 6–7 where they undergo medical checkups including ophthalmic tests under the School Health Act^21^. Therefore, those who have not been diagnosed with ophthalmic abnormalities before this age can be diagnosed at this time, which may contribute to the highest prevalence at this age.

The prevalence of strabismus among children born at term ranges from 1 to 3% worldwide^7,22–24^. Meanwhile, the prevalence of strabismus in preterm children ranged from 16 to 22%^25–27^. There have been several single institution-based studies in Korea reporting that the prevalence of strabismus among premature infants was 36.6% at 3 years of age^28^ and 16.0% at 2 years of age^29^. In cases of ROP, 80% of children with a history of severe ROP and 60% of children with Type I ROP were reported to develop strabismus during the first 6 years of life^9^. In addition, a study investigating ROP among premature infants with a GA ≤ 32 weeks showed that ROP cases that were treated had a 2.06 and 2.27 times higher risk of developing strabismus than mild and moderate cases of ROP, respectively^10^. Although the prevalence of strabismus among patients with ROP was lower in the present study, the higher risk of developing strabismus in cases of ROP and tROP was consistent with previous studies. It was reported that the increase in strabismus in ROP could be attributed to higher neurological deficits in children with ROP, refractive error, or loss of visual function due to cicatrical ROP which is related to structural changes, especially in severe ROP^8,27,30^.

Amblyopia has been reported in 0.8% to 2.6% of children aged 72 months or less in various countries^22–24,31^. Among infants with modestly low BW (1500–2499 g), 7.3% were diagnosed with amblyopia at the age of 6 years in an Australian population-based study^32^. Among Korean children born preterm (GA < 34 weeks or BW < 2000 g), 11.0% were diagnosed with amblyopia at the age of 3 years. It is challenging to compare the prevalence of amblyopia in the present study with those reported in previous reports due to the differences in the inclusion criteria, though, the prevalence of 4.09% among patients with ROP and 15.6% among those with tROP at the age of 9 years in the present study provides the longest follow-up among population-based studies. The higher risk of developing amblyopia in patients with ROP and tROP should be understood in consideration with refractive abnormalities or strabismus.

Pediatric glaucoma can result from various distinct pathologies^33^. The incidence of childhood glaucoma was reported to be 2.29 out of 100,000 residents younger than 20 years old in the United States^34^. In preterm children, glaucoma was observed in 0.11% (3/2660) of the infants with very low BW (VLBWIs, BW < 1500 g) born between 2013 and 2014 at the corrected age of 18–24 months, according to a report from Korean Neonatal Network (KNN) database that represents 85% of all VLBWIs in Korea^35^. The prevalence of glaucoma among those with ROP has been reported as 1.67% in the advanced stages of ROP at 6 years of age in the ETROP study^19^, and as 1.36% (diagnosed before 66 months) in a large Indian cohort^36^. Glaucoma development in association with ROP treatment also has been reported in 0.1% of patients with ROP who underwent laser photocoagulation^37^, and 6% of patients with ROP who underwent vitreoretinal surgery, all of which were diagnosed before 24 months^38^. In the present study, glaucoma prevalence among patients with ROP at the 6th year was 1.49% and among those with tROP at the 2nd year was 5.43%, which is comparable to the previous reports.

There have been many studies regarding refractive problems related to preterm birth and ROP. Preterm birth is a known risk factor for refractive problems^17,28,30,39^, such as hyperopia and astigmatism^17^, or myopia^30,39^. It is known that the incidence and severity of myopia increases with the severity of ROP^12,13^. Increased risks of myopia in infants treated with the laser for severe ROP have also been reported^11^. In the present study, we included the diagnostic codes for myopia or hyperopia as refractive abnormalities. Clinicians are less likely to register these diagnostic codes when there is mild myopia or hyperopia. Therefore, most cases with registered refractive diagnostic codes can roughly be regarded as having moderate or severe abnormalities. The prevalence reported in this study should be regarded as a ballpark figure. However, it is worth noting that the risk of refractive abnormalities was higher in the presence of ROP or treatment for ROP in the present study, in accordance with previous studies.

The strength of the present study is that the largest nationwide population-based database was used to investigate long-term annual ophthalmic outcomes during a 10-year follow-up period. Furthermore, it is unlikely that there were missing ROP cases, as all premature infants need admission to hospitals. Therefore, the diagnostic code for ROP is likely to be properly registered. In addition, to determine the risk of ophthalmic complications according to the presence of ROP in premature infants, “premature infants without ROP” was set as a reference group to analyze HRs. The performance of ROP treatment was considered to depend on the severity of ROP. Therefore, this study also analyzed the extent of ophthalmic complications risk in severe ROP, that is, in those treated for ROP, by calculating HRs using “ROP infants who did not undergo treatment for ROP” as a reference group.

There were several limitations to the current study. First, the cases in the NHI database can only be identified by diagnostic or procedure codes, which are registered by clinicians. Therefore, visual function (visual acuity data) could not be analyzed in the present study. Second, detailed diagnostic codes may have not been correctly registered by clinicians. We tried to include as many detailed codes for each complication as possible with the assumption that the diagnostic codes in ROP during childhood are related to ROP or treatment for ROP (e.g., the diagnostic code of “Senile cataract” in childhood cannot be "senile,” and is more likely to be cataract after ROP). However, there remains the possibility that the registered codes for each complication was not only for the complication of ROP but due to other causes such as trauma. Third, since each eye cannot be evaluated separately using the NHI database, complications related to inter-eye differences could not be investigated in the present study. Ophthalmic complications caused by the inter-eye differences, such as anisometropic amblyopia or strabismus, need to be investigated in future studies. Lastly, there could have been a detection bias in the young age group during follow-up. In particular, patients treated for ROP or those with severe ROP are more likely to attend clinics, and the complications are more likely to be detected at a younger age. Otherwise, the complications are more likely to be diagnosed when the child can express their symptoms or during school age Therefore, the incidence or prevalence, especially in young infants, should be interpreted in consideration of this detection bias. However, even considering this, the prevalence of complications in the 10th year could be considered to be reliable.

In conclusion, the present study reported the nationwide epidemiology of ophthalmic complications related to ROP in South Korea using a population-based database over a 10-year follow-up period. The three most common ophthalmic complications of ROP during the first decade of life in South Korea are strabismus, amblyopia, and glaucoma. There was a significantly higher risk of ophthalmic complications in the presence of ROP and treatment for ROP. Finally, these findings may shed light on our understanding of the complications of ROP in childhood and will help develop national management plans for ROP in premature infants.

## Data availability

The data that support the findings of this study are available from the corresponding author upon reasonable request.

## Supplementary Information

Supplementary Information.
