# Supplementary material for: Ophthalmic complications in retinopathy of prematurity in the first decade of life in Korea using the national health insurance database
Source: Sci Rep. 2022 Jan 18;12:911. doi: 10.1038/s41598-021-04616-7 (PMC8766475; doi:10.1038/s41598-021-04616-7)

Ophthalmic Complications in Retinopathy of Prematurity in the First Decade of Life in Korea using the National Health Insurance Database

Eun Hee Hong[1, +], Yong Un Shin[1, +], Gi Hwan Bae[2], Young Jin Choi[3], Seong Joon Ahn[1], Inah Kim[2, *], Heeyoon Cho[1, *]

[1]Department of Ophthalmology, Hanyang University College of Medicine, Seoul, Korea

[2]Department of Occupational and Environment Medicine, Hanyang University College of Medicine, Seoul, Korea

[3]Department of Pediatrics, Hanyang University College of Medicine, Seoul, Korea

*Corresponding Authors: Heeyoon Cho, MD, PhD, hycho@hanyang.ac.kr
*Co-corresponding Author: Inah Kim, MD, MPH, PhD, inahkim@hanyang.ac.kr
[+]These authors (Eun Hee Hong and Yong Un Shin) contributed equally to this work.

Supplementary Table S1. KCD-6 codes for congenital anomalies and perinatal injuries that might affect normal development.*

**Description**	**KCD-6 Codes**
Anencephaly	Q00.0-2
Encephalocele	Q01.0-2,8
Microcephaly	Q02
Congenital hydrocephalus	Q03
Other congenital malformations of the brain	Q04.0-9
Other disturbances of the cerebral status of newborns	P91
Disorders of muscle tone of newborns	P94
Birth trauma	P10-15
Intracranial non-traumatic hemorrhage of the fetus and newborn	P52
Intrauterine hypoxia	P20
Birth asphyxia	P21

KCD-6, Korean Classification of Disease, sixth edition

*KCD-6 is a modified version of the International Classification of Diseases, 10th edition (ICD-10).

Supplementary Table S2. KCD-6 codes for ophthalmic complications.*

Description	KCD-6 Codes
Amblyopia	
amblyopia	H53.0
Cataract	
congenital cataract	Q12.0
senile cataract	H25
infantile, juvenile, and presenile cataract	H26.0
other specified cataracts	H26.8
unspecified cataract	H26.9
other disorders of the lens in diseases classified elsewhere	H28
Glaucoma	
congenital glaucoma	Q15.0
glaucoma	H40
glaucoma in diseases classified elsewhere	H42
Nystagmus	
nystagmus and other irregular eye movements	H55
Strabismus	
paralytic strabismus	H49
other strabismus	H50
other disorders of binocular movement	H51
Retinal detachment	
retinal detachment with a retinal break	H33.0
retinoschisis and retinal cysts	H33.1
serous retinal detachment	H33.2
tractional retinal detachment	H33.4
and other retinal detachments	H33.5

Refractive abnormalities	
hyperopia	H52.0
myopia	H52.1
degenerative myopia	H44.2
Cataract surgery	
phacoemulsification	S5119
pars plana lensectomy	S5110
extracapsular or intracapsular extraction	S5111
intraocular lens implantation	S5116-7

KCD-6, Korean Classification of Disease, sixth edition

*KCD-6 is a modified version of the International Classification of Diseases, 10th edition (ICD-10).

Supplementary Table S3. The number of annual incident cases and annual incidence rate of ophthalmic complications in patients with ROP and those treated for ROP (tROP) at each year of life according to the GA.

	Overall (GA < 37wks)				GA < 28wks				28wks ≤ GA < 37wks			
	ROP		tROP		ROP		tROP		ROP		tROP	
**Total, N**	6995		276		284		75		6711		201	
	Incident cases, N	Annual incidence, %	Incident cases, N	Annual incidence, %	Incident cases, N	Annual incidence, %	Incident cases, N	Annual incidence, %	Incident cases, N	Annual incidence, %	Incident cases, N	Annual incidence, %
**Age**												
**~1 y**	561	8.02%	91	32.97%	34	11.97%	13	17.33%	527	7.85%	78	38.81%
**1-2 y**	186	2.66%	15	5.43%	11	3.87%	4	5.33%	175	2.61%	11	5.47%
**2-3 y**	161	2.30%	21	7.61%	12	4.23%	7	9.33%	149	2.22%	14	6.97%
**3-4 y**	168	2.40%	16	5.80%	15	5.28%	6	8.00%	153	2.28%	10	4.98%
**4-5 y**	225	3.22%	19	6.88%	9	3.17%	5	6.67%	216	3.22%	14	6.97%
**5-6 y**	241	3.45%	17	6.16%	9	3.17%	5	6.67%	232	3.46%	12	5.97%
**6-7 y**	192	2.74%	16	5.80%	13	4.58%	7	9.33%	179	2.67%	9	4.48%
**7-8 y**	224	3.20%	7	2.54%	6	2.11%	3	4.00%	218	3.25%	4	1.99%
**8-9 y**	183	2.62%	3	1.09%	8	2.82%	2	2.67%	175	2.61%	1	0.50%
**9-10 y**	159	2.27%	6	2.17%	7	2.46%	2	2.67%	152	2.26%	4	1.99%

GA, Gestational age; ROP, retinopathy of prematurity

Supplementary Table S4. The number of annual incident cases and annual incidence rate of each ophthalmic complication and refractive errors in patients with ROP and those treated for ROP (tROP) at each year of life.

	**Amblyopia**				**Cataract**				**Glaucoma**			
	**ROP**		**tROP**		**ROP**		**tROP**		**ROP**		**tROP**	
**Total, N**	6995		276		6995		276		6995		276	
	Incident cases, N	Annual incidence, %	Incident cases, N	Annual incidence, %	Incident cases, N	Annual incidence, %	Incident cases, N	Annual incidence, %	Incident cases, N	Annual incidence, %	Incident cases, N	Annual incidence, %
**Age**												
**~1 y**	219	3.13%	18	6.52%	27	0.39%	14	5.07%	83	1.19%	49	17.75%
**1-2 y**	31	0.44%	2	0.72%	13	0.19%	3	1.09%	13	0.19%	5	1.81%
**2-3 y**	40	0.57%	10	3.62%	23	0.33%	5	1.81%	27	0.39%	4	1.45%
**3-4 y**	48	0.69%	7	2.54%	25	0.36%	3	1.09%	30	0.43%		0.00%
**4-5 y**	121	1.73%	11	3.99%	9	0.13%	3	1.09%	59	0.84%	6	2.17%
**5-6 y**	149	2.13%	19	6.88%	14	0.20%	1	0.36%	74	1.06%	3	1.09%
**6-7 y**	163	2.33%	26	9.42%	2	0.03%	2	0.72%	81	1.16%	10	3.62%
**7-8 y**	130	1.86%	9	3.26%	6	0.09%	2	0.72%	109	1.56%	6	2.17%
**8-9 y**	113	1.62%	9	3.26%	5	0.07%		0.00%	94	1.34%		0.00%
**9-10 y**	89	1.27%	11	3.99%	1	0.01%	1	0.36%	92	1.32%	2	0.72%

	**Nystagmus**				**Strabismus**				**Retinal detachment**			
	**ROP**		**tROP**		**ROP**		**tROP**		**ROP**		**tROP**	
**Total, N**	6995		276		6995		276		6995		276	
	Incident cases, N	Annual incidence, %	Incident cases, N	Annual incidence, %	Incident cases, N	Annual incidence, %	Incident cases, N	Annual incidence, %	Incident cases, N	Annual incidence, %	Incident cases, N	Annual incidence, %
**Age**												
**~1 y**	3	0.04%	1	0.36%	268	3.83%	21	7.61%	20	0.29%	16	5.80%
**1-2 y**	2	0.03%	1	0.36%	184	2.63%	21	7.61%	1	0.01%	1	0.36%
**2-3 y**	3	0.04%	2	0.72%	111	1.59%	10	3.62%		0.00%		0.00%

**3-4 y**	2	0.03%	1	0.36%	113	1.62%	13	4.71%		0.00%		0.00%
**4-5 y**	5	0.07%	3	1.09%	123	1.76%	19	6.88%		0.00%		0.00%
**5-6 y**	3	0.04%	1	0.36%	95	1.36%	16	5.80%		0.00%		0.00%
**6-7 y**	1	0.01%		0.00%	82	1.17%	9	3.26%		0.00%		0.00%
**7-8 y**	5	0.07%	3	1.09%	88	1.26%	8	2.90%	2	0.03%		0.00%
**8-9 y**	4	0.06%	1	0.36%	81	1.16%	5	1.81%	1	0.01%		0.00%
**9-10 y**	4	0.06%	1	0.36%	70	1.00%	5	1.81%		0.00%		0.00%

	**Cataract surgery**				**Refractive abnormalities**			
	**ROP**		**tROP**		**ROP**		**tROP**	
**Total, N**	6995		276		6995		276	
	Incident cases, N	Annual incidence, %	Incident cases, N	Annual incidence, %	Incident cases, N	Annual incidence, %	Incident cases, N	Annual incidence, %
**Age**								
**~1 y**	13	0.19%	13	4.71%	78	1.12%	11	3.99%
**1-2 y**		0.00%		0.00%	111	1.59%	17	6.16%
**2-3 y**		0.00%		0.00%	163	2.33%	18	6.52%
**3-4 y**		0.00%		0.00%	381	5.45%	32	11.59%
**4-5 y**		0.00%		0.00%	496	7.09%	32	11.59%
**5-6 y**		0.00%		0.00%	519	7.42%	19	6.88%
**6-7 y**	1	0.01%	1	0.36%	545	7.79%	16	5.80%
**7-8 y**		0.00%		0.00%	618	8.83%	22	7.97%
**8-9 y**		0.00%		0.00%	554	7.92%	9	3.26%
**9-10 y**		0.00%		0.00%	368	5.26%	11	3.99%

ROP, retinopathy of prematurityy

Supplementary Figure S1. The annual cumulative incidence of ophthalmic complications at each year of life among ROP (A) and those treated for ROP (B). "Total" represents the cases with at least one of the complications (amblyopia, cataract, glaucoma, nystagmus, retinal detachment (RD), and strabismus). ROP = retinopathy of prematurity

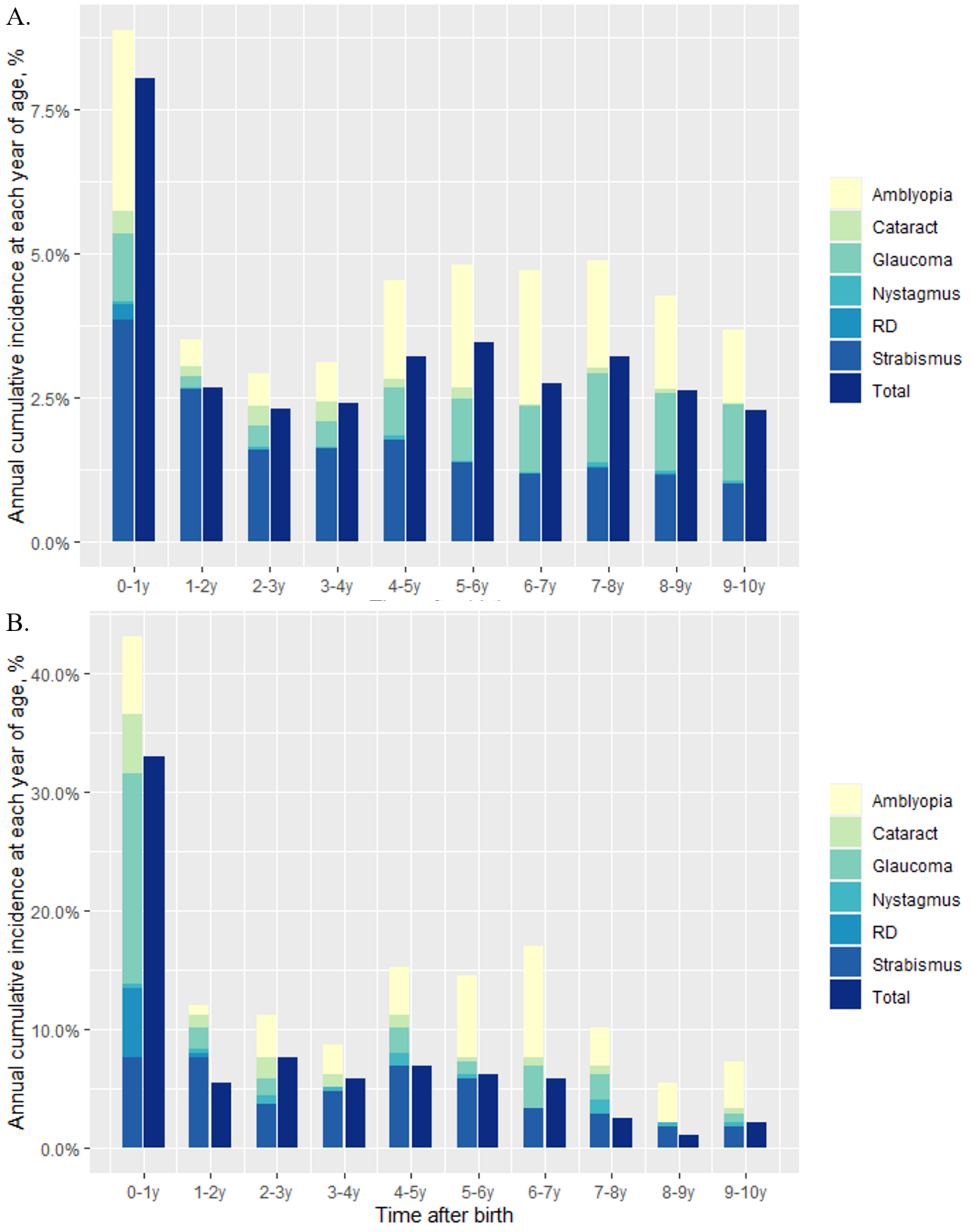

Supplementary Figure S2. The hazard ratio plots of ophthalmic complications according to the presence of ROP among premature infants. (A) Amblyopia, (B) cataract, (C) glaucoma, (D) nystagmus, (E) strabismus, and (F) retinal detachment. ROP = retinopathy of prematurity

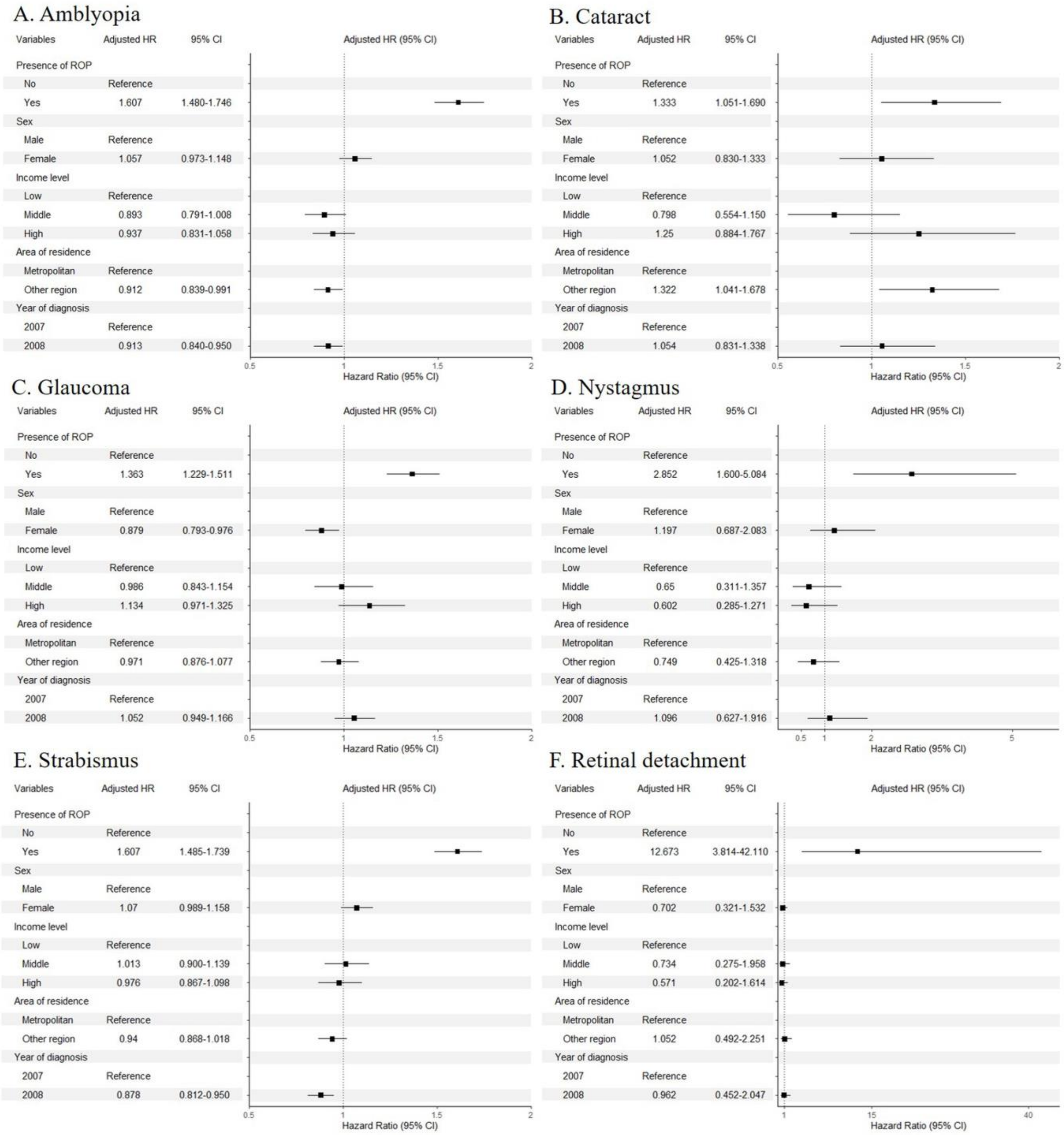

Supplementary Figure S3. The hazard ratio plots of ophthalmic complications according to the presence of treatment for ROP among ROP infants. (A) Amblyopia, (B) cataract, (C) glaucoma, (D) nystagmus, (E) strabismus, and (F) retinal detachment. ROP = retinopathy of prematurity.

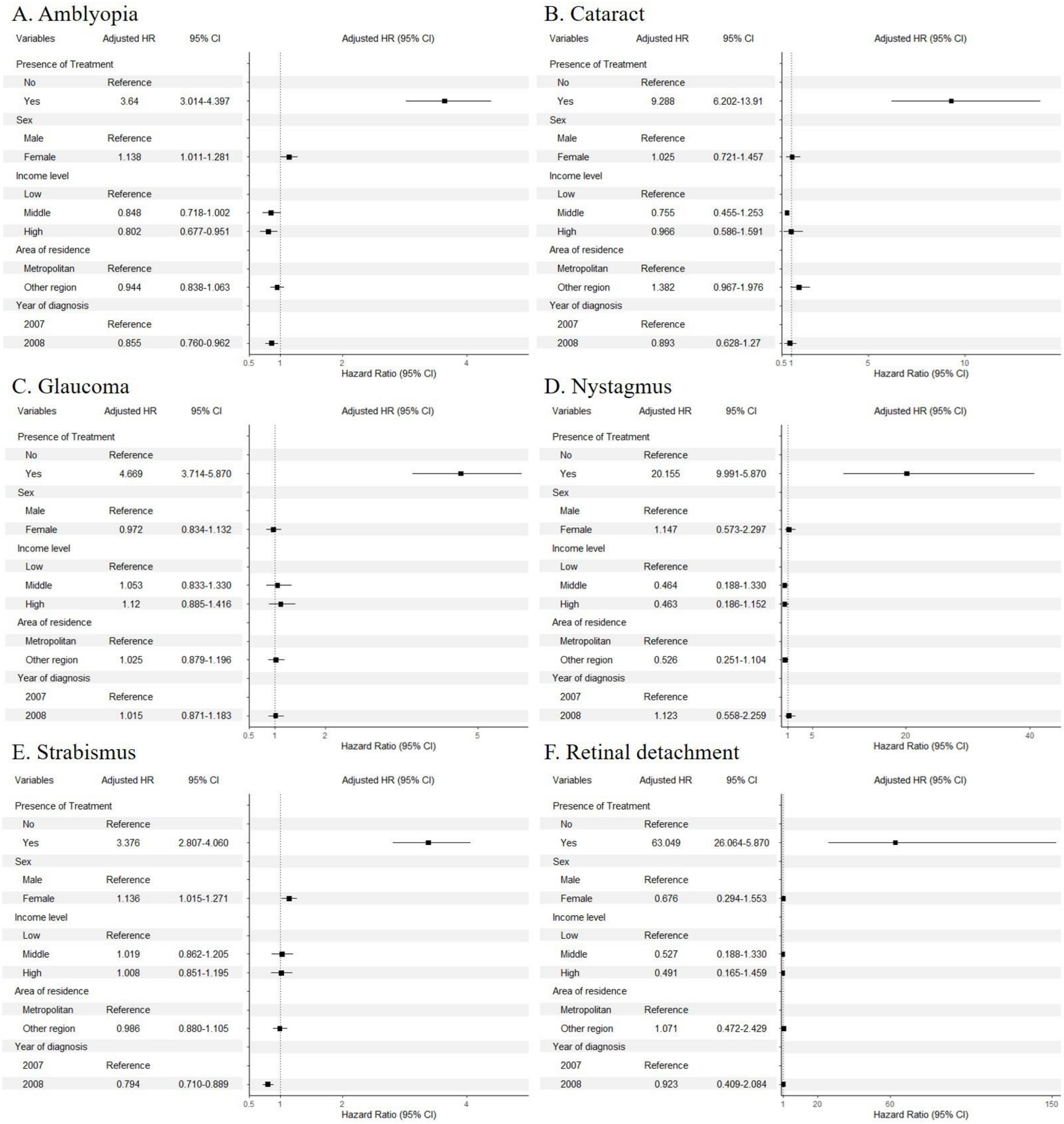

Supplementary Figure S4. The epidemiology of refractive abnormalities (myopia, hyperopia) in patients with ROP and those treated for ROP. The period prevalence at each year of life among patients with ROP (A) and those treated for ROP (B), the hazard ratio plots according to the presence of ROP among premature infants (C) and according to the performance of ROP treatment among ROP infants (D). ROP = retinopathy of prematurity

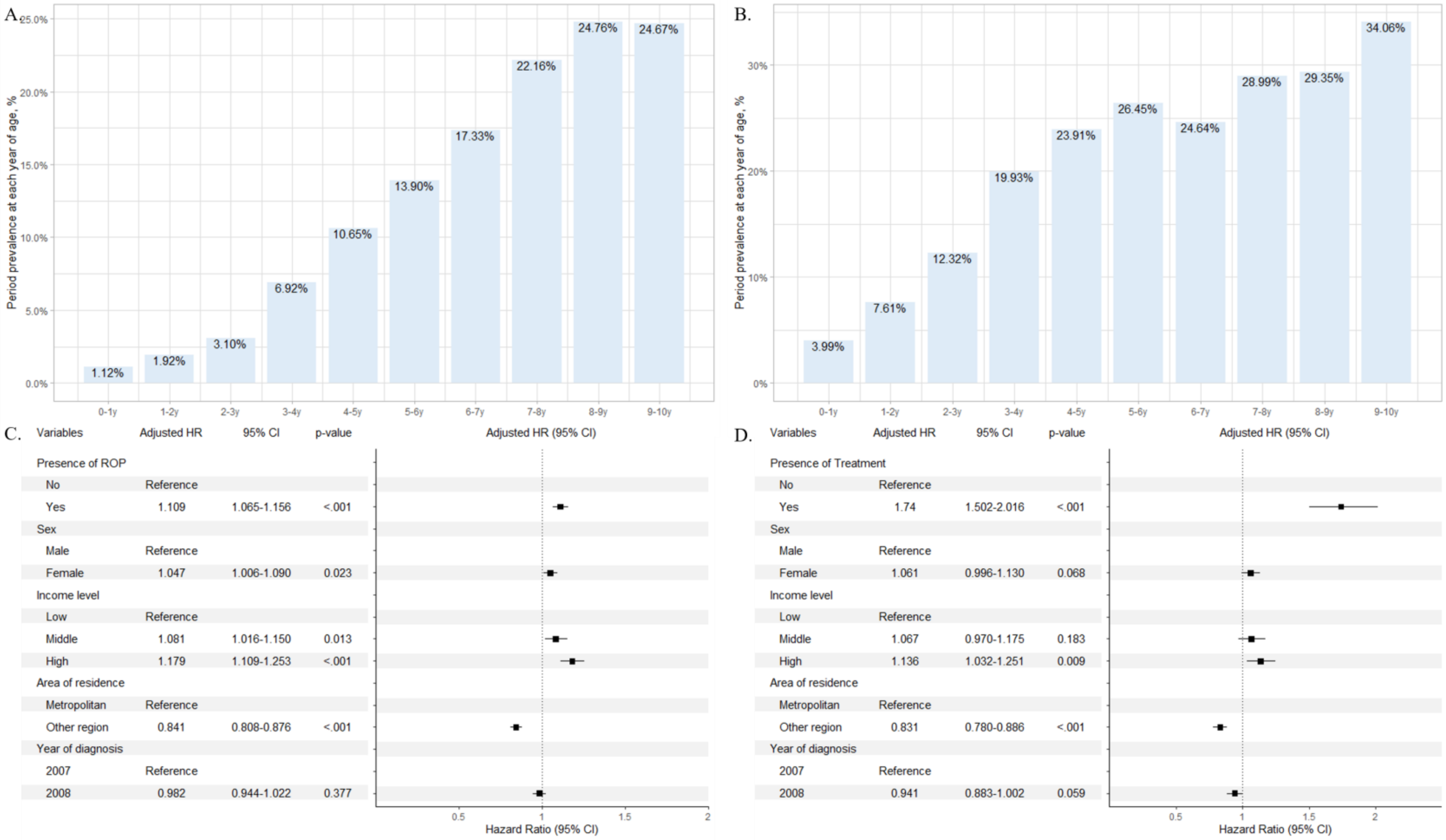

Supplement: Supplementary file 1 — Supplementary Information. [file 41598_2021_4616_MOESM1_ESM.pdf]
